# Factors That Prevent Mosquito-Borne Diseases among Migrant Workers in Taiwan: Application of the Health Belief Model in a Church-Based Health Promotion Study

**DOI:** 10.3390/ijerph19020787

**Published:** 2022-01-11

**Authors:** Yu-Shan Tai, Hao-Jan Yang

**Affiliations:** 1 Department of Public Health, Chung Shan Medical University, No. 110 Sec. 1 Jianguo N. Rd., Taichung 40201, Taiwan; taisun7788@gmail.com; 2 Department of Family and Community Medicine, Chung Shan Medical University Hospital, No. 110 Sec. 1 Jianguo N. Rd., Taichung 40201, Taiwan

**Keywords:** foreign migrant workers, health belief model, mosquito-borne disease

## Abstract

Background: Southeast Asian countries have long been considered epidemic areas for mosquito-borne diseases (MBDs), and most imported cases of infectious diseases in Taiwan are from these areas. Taiwanese migrant workers are mainly of Southeast Asian nationality, and of these, 22% are Filipino. Migrant workers’ knowledge of MBDs and self-protection behaviors are beneficial to disease prevention and treatment. This study aims to understand the effectiveness of a health education intervention (HEI) for Filipino migrant workers in Taiwan and explores the factors affecting preventive practices. Methods: The study was conducted between May to September 2018. Participants were recruited from two Catholic churches in Taichung City. A professional delivered a 30 min HEI in person, and a structured questionnaire was used to acquire and assess participants’ knowledge, health beliefs, and preventive behaviors for MBDs before and after the intervention. Results: A total of 291 participants were recruited. The intervention program showed a positive impact on the migrant worker’s knowledge and the perceived severity, perceived benefits, perceived barriers, and preventive practices. Knowledge, perceived severity, and perceived barriers were factors influencing preventive practices in Filipino migrant workers. Conclusions: The results of this study demonstrated that we can direct our efforts towards three areas: improving foreign migrant workers’ awareness of diseases, emphasizing the severity of the disease, and eliminating possible hindrances in the future. As one example, migrant workers could be proactively provided with routine medical examinations and multilingual health education lectures to improve knowledge and preventive practices to contain the spread MBDs.

## 1. Background

Mosquito-borne diseases (MBDs) are diseases transmitted to humans through bites of infected and contagious mosquitoes. These include dengue fever, malaria, Chikungunya fever, Zika virus infection, and yellow fever, among others [1]. In recent years, the number of foreign migrant workers in Taiwan has grown significantly. The migrant workers are mainly from Southeast Asian countries: 38% are from Indonesia, followed by 31% from Vietnam, and 22% from the Philippines, and 9% from Thailand, Malaysia, and Mongolia [2]. Mosquito vectors of infectious diseases are widespread throughout Southeast Asian countries, and Southeast Asia is the main source area of imported dengue fever cases in Taiwan [3]. In 2017, the confirmed rate of notifiable infectious diseases obtained by the international port and border quarantine was as high as 65% in foreign migrant workers [4]. Therefore, it is an important and urgent matter to provide health education for migrant workers on MBD. However, due to the language barrier in communication, it is not easy for health agencies to fully implement health education interventions (HEIs) for foreign migrant workers. English is the mother tongue in the Philippines, and English is also the most commonly spoken foreign language in Taiwan. Therefore, Filipino migrant workers are a relatively easy group of people to communicate with.

The most prevalent MBDs in the Philippines are dengue fever and malaria. There were over 400,000 cases of dengue fever in 2019, which caused 1500 deaths in the Philippines [5]. In 2019, Taiwan had a total of 540 cases of dengue fever imported from abroad, of which 81 cases (15%) were from the Philippines; in 2020, there were 64 cases of dengue fever imported from abroad, including 13 cases (20%) from the Philippines [6]. As for malaria, there were more than 30,000 malaria cases in the Philippines in 2019 [7]. Taiwan was certified a malaria-free country by the World Health Organization (WHO) in 1965. Cases of malaria are mainly imported from abroad. There are about 10 to 30 imported cases each year, mainly infected individuals arriving from Southeast Asia, Africa, and Oceania [1].

Current medical examinations for foreign migrant workers in Taiwan do not include malaria blood smears or checkups for dengue fever. The prevention and control of MBDs rely on self-protection and early medical treatment. However, the countries of origin of most foreign migrant workers have poor sanitary conditions, and the concept of disease prevention is less widely known. The living habits and perceptions and concepts of health and diseases are also different from the general population in Taiwan. Therefore, when there are cases of mosquito-borne infections, the risk of disease spread and the clustering of infectious diseases will increase. A 2017 study from Singapore found that migrant workers have a higher risk of infectious diseases than native people. This may be due to the higher prevalence of the diseases in their country of origin, socioeconomic factors, language and cultural barriers, local living conditions, and imparity of healthcare services, or other factors [8]. The extended personal and sociocultural disadvantages are stumbling blocks that hinder the formation and development of individual health beliefs, thereby increasing the risk of disease.

The health belief model (HBM) includes concepts of perceived: susceptibility, severity, benefits, and barriers [9]. Nowadays, this model is often used to explain health-related behaviors and the contribution of hygiene education. The HBM can not only be applied to influenza prevention behaviors and related factors for the local residents of Taiwan [10], but it is also applicable to Taiwanese immigrants. For example, a 2014 study on malaria prevention behaviors adopted by Taiwanese immigrants when returning home pointed out that the perceived: susceptibility, severity, and benefits were factors that affected the participants’ malaria prevention behaviors [11]. In addition, the HBM can also be used to evaluate the effectiveness of interventions. After Malaysian high school students received HEIs for dengue fever, students’ knowledge scores significantly improved [12].

This study proposed the design of a set of HEIs for Filipino migrant workers in Taiwan and used the HBM as the basis to explore the extent to which this intervention changed migrant workers’ awareness, beliefs, and preventive behaviors to avoid mosquito vectors of infectious disease. Additionally, we elucidated the factors that influence the awareness and health beliefs of preventive behaviors to serve as a reference for future strategies for effective disease prevention.

## 2. Methods

### 2.1. Research Design and Research Participants

This study adopted a one-group pre-test–post-test design to evaluate the effectiveness of a HEI for migrant workers from the Philippines. The HEI refers to the information publicized by the Taiwan Centers for Disease Control (TCDC). Our research team screened the health education materials related to dengue fever and malaria and designed 10–20 min of health education content. The implementation of the health education was carried out by two assistants on our research team who were familiar with the prevention and treatment of infectious diseases and were fluent in English. In-person lectures and videos were used to deliver the information. Evaluation of the HEI was measured by a structured questionnaire (Supplementary Materials S1) using a paper and pencil assessment designed by the research team. A pre-test was conducted before attending the HEI, and a post-test was given after the completion of the health education course. Pre-test and post-test each took 5–10 min. The contents of the pre-test and post-test questionnaires were the same and we examined the difference before and after the intervention of the health education course.

Participants of this study were mainly migrant workers from the Philippines as the official language there is English, which reduces the language barrier. It is not easy to contact migrant workers in Taiwan, thus as most Filipino migrant workers are Catholic and attend the Sunday Mass, the recruitment sites were set up at two Catholic churches in Taichung City (each church had approximately 400 Filipino migrant workers). Research assistants explained the research using the spare time before and after the Sunday Mass, eligible members were recruited, and written consent was obtained from all participants before the HEI was implemented. A pre-test and post-test were conducted before and after the intervention program. The whole process took about 30 min. The criteria for participant inclusion were: (1) over 20 years of age, (2) able to communicate in English, and (3) Filipino migrant workers who were willing to participate in the study. Exclusion criteria were those who did not fully complete pre- and post-test surveys or the health education program.

### 2.2. Measurements

This study used a self-compiled structured questionnaire as an assessment tool. The design of the questionnaire is based on the HBM. In addition, after consulting with domestic and foreign literature, we found that the degree of understanding of MBDs was correlated to the individual’s preventive practices. Therefore, we added knowledge to the questionnaire as a variable and explored the relationship between preventive practices and variables such as knowledge and health beliefs. The contents of the questionnaire included the demographic characteristics of the participant and their knowledge, cues to action, health beliefs, and preventive behaviors towards MBDs. Among these, the higher the knowledge score on MBD was, the better the understanding of dengue fever and malaria. Cronbach’s α value for knowledge domain was 0.66. The health beliefs of MBDs were subdivided into perceived susceptibility, perceived severity, perceived benefits, and perceived barriers. These were all scored based on a 5-point Likert scale. The higher the score, the higher the degree of belief is on the subscales of perceived susceptibility, perceived severity, perceived benefits, and perceived barriers. The Cronbach’s α value of the health belief scale was 0.78. The mosquito vector infectious disease prevention behavior scale was scored based on a 5-point Likert Scale. The higher the score is, the better the preventive practices are. The Cronbach’s α value was 0.90.

The health education materials of this study consulted the general public and the professional version of the English global information website of the TCDC and the English website of the WHO. Information and literature on dengue fever and malaria-related epidemics and health education were compiled and printed. The contents included the cause of the disease, the mode of transmission, symptoms, treatment and care, and prevention methods.

### 2.3. Data Analysis

After collecting and sorting the questionnaires, results of the valid questionnaires were input into Microsoft Excel 2016, and the statistical software SPSS 23.0 (IBM Corporation, Armonk, NY, USA) and AMOS 24.0 (IBM Corporation, Armonk, NY, USA) were used for statistical analysis. The distribution of basic demographic data, cues to action, knowledge of MBD, health beliefs, and prevention practices were demonstrated by frequency distribution, percentage, mean, and standard deviation. In order to assess the impact of the HEI, a paired sample *t*-test was used to confirm whether there was a significant difference between the pre- and post-tests in the areas of knowledge, health beliefs, or preventive practices. A path analysis diagram was used to show the same survey conducted at different times. Knowledge, health beliefs, and preventive practices towards MBDs were observation variables, and we sought to determine the differences before and after the HEI. Lastly, using the path analysis, a diagram was plotted to examine the effects of knowledge, health beliefs, and other variables on the participants’ disease-prevention behaviors.

## 3. Results

Among the 291 Filipino migrant workers recruited in this study, women accounted for the majority (*n* = 228, 78.4%) (Table 1). The majority were either 25–29 years old (39.5%) or 30–34 years old (26.5%). About 66.3% of the participants were high school graduates. More than half of the research participants mentioned that it was their first time in Taiwan (59.1%), but 44% of the participants had been in Taiwan for a total of 2–4 years. The manufacturing industries accounted for the majority of their field of work (91.4%). About 60.1% of the research participants understood and spoke Chinese. More than half of the participants earned less than 20,000 New Taiwan Dollar (NTD, 1 NTD = 0.036 USD) a month. In terms of cue to action, the main source of information on MBDs was television (50.9%), and the main human sources of information were medical personnel (44.7%) and family members (44.3%). Eighteen participants (6.2%) said that they had contracted mosquito-borne infections, and 38 (13.1%) said that their family members had suffered from MBDs.

Regarding the knowledge of MBDs, 94.2% of the study subjects knew that “dengue fever is transmitted by mosquitoes”; 90% of the participants understood that “if they have any symptoms, they should seek medical help and inform the doctor of their history of travel”. These two items had high rates of correct answers (Table 2). The items that had lower correct answer rates were “drinking unsafe water and eating raw food will result in malaria infections” (15.8%) and “all MBDs are caused by dengue virus” (16.8%). This showed that migrant workers had limited knowledge about the mechanisms of infectious diseases. However, after the HEI, the correct answer rate of these two items increased to 74.9% and 60.5%, respectively. The two items were statistically significant (*p* < 0.05). The accuracy of all 20 knowledge items was improved after the health education program. However, the improvement of three of the items (1, 9, 17) did not reach the level of significance.

All items of the health belief subscales improved after the HEI (Table 2). The two items on perceived susceptibility were scored high, but the difference in improvement in the post-test compared to the pre-test was not significant. Among the six items for perceived severity, the improvement of four items showed a significant difference in the post-test compared to the pre-test (*p* < 0.0001). Among the five items on perceived benefits, two of the items showed significant improvements in the post-test compared to the pre-test (*p* < 0.0001). Four out of seven items on perceived barriers were significantly reduced after the HEI. All items on preventive practices were significantly higher in the post-test than the pre-test (*p* < 0.0001). After summing the scores of the knowledge and health beliefs dimensions (Table 3), the knowledge of MBDs shown by the research participants was significantly improved (paired *t* = −27.00, *p* < 0.0001). The effect of health education on the improvement of perceived susceptibility was not significant (paired *t* = −0.60, *p* > 0.05), but showed significant improvements in perceived severity (paired *t* = −6.64, *p* < 0.0001), perceived benefit (paired *t* = −0.60, *p* > 0.05. *t* = −8.80, *p* < 0.0001), preventive practices (paired *t* = −11.09, *p* < 0.0001), and a substantial decrease in perceived barriers (paired *t* = 2.38, *p* = 0.018).

**Figure 1:**
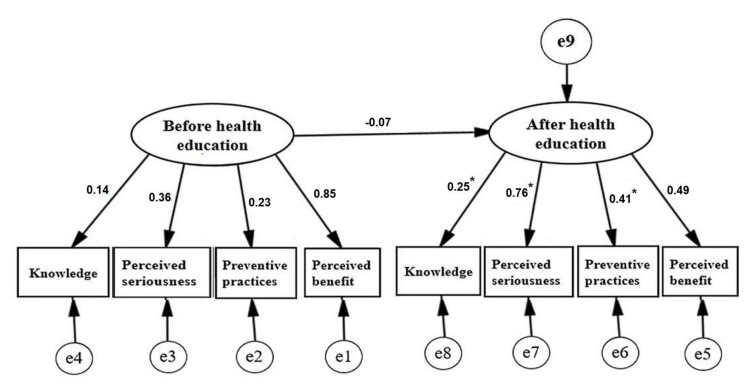
Path analysis for knowledge, perceived seriousness, perceived benefit, and preventive practice from before to after implementing the health education program. Note: The numbers in the figure represent path coefficients, the parameters that affect the size of the independent variable on the dependent variable. e1–e9: measurement errors. * *p* < 0.05.

Furthermore, this study used AMOS 24.0 software to conduct a path analysis to explore the differences before and after HEI. Since the perceived susceptibility was not significantly affected by the HEI in the initial analysis and the model fit was not adequate after it was included in the analysis, only the mosquito-borne infectious disease knowledge, perceived severity, perceived benefits, prevention practices, and other variables were included in the path analysis. The results are shown in Figure 1. After the HEI, the path coefficients of knowledge, perceived severity, and preventive practices reached statistical significance compared to pre-HEI. The path coefficients also improved.

**Figure 2:**
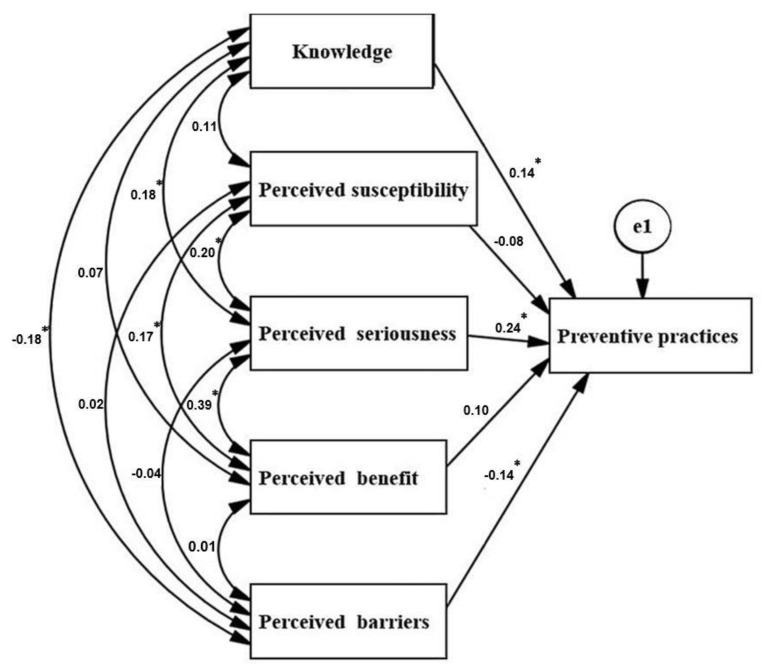
Knowledge and health belief predictors of preventive practice. Note: The numbers in the single-headed arrows represent path coefficients, the parameters that affect the size of the independent variable on the dependent variable. e1: measurement error. A double-headed, curved arrow indicates that variables are correlated, numbers representing correlation coefficients. * *p* < 0.05.

In order to understand the relevant factors that influence preventive practice, we took preventive practice as an outcome, and use a path analysis to examine the impact of knowledge and all aspects of health beliefs (Figure 2). The results suggested that the factors that drove migrant workers to adopt preventive practices were “knowledge” (β = 0.14, *p* = 0.01), “perceived severity” (β = 0.24, *p* < 0.001), and “perceived barriers” (β = −0.14), *p* = 0.009), while the perceived susceptibility and perceived benefits did not directly affect the preventive practices, but still had an indirect impact on the preventive practice through the perceived severity (βs with perceived susceptibility and perceived benefit were 0.20 and 0.39, respectively).

## 4. Discussion

This study is the first to explore the prevention of mosquito vector infections in foreign migrant workers in Taiwan. The results demonstrated that brief HEIs can effectively increase the knowledge and health beliefs and improve preventive practices in groups of migrant workers from Southeast Asia where MBDs are prevalent. The improvement of knowledge, perceived: severity, benefits, and barriers were all essential factors for predicting preventive practice.

Consistent with the results of most previous studies [13,14,15], this study found that about half of Filipino migrant workers obtain information about MBDs from TV. Compared with other sources of information, television is a more popular mass media. Health information promotion through this approach is more effective for migrant workers. In addition, nearly half of the study participants received infectious disease-related information from two sources: medical personnel and family members. This result indicates that, whether through routine physical examinations or an outpatient environment, if the first-line medical staff can provide migrant workers with information about health and education related to infectious diseases, this will help improve their knowledge and health beliefs. Since family members may also be approached to communicate medical-related information, we can expect that if migrant workers in Taiwan acquire sufficient knowledge and beliefs, they will be able to benefit their families in the Philippines when they return to their home country.

Since medical personnel play an important role in migrant workers’ cues to action, it is not difficult to understand that after the HEIs lead by researchers with medical-related training backgrounds, migrant workers’ knowledge, health beliefs, and preventive practices towards mosquito-borne infections significantly improved. The improvement in knowledge and preventive practice was particularly obvious, which is consistent with other recent studies [12,16]. The only exception was the perceived susceptibility aspect of health beliefs. It did not show a significant difference before and after the HEI. The scores of the pre- and the post-tests were high, indicating that the participants did not think that the risk of getting the disease was high, even after the explanation and education from experts. This may be explained by the fact that most of the migrant workers participating in the study had no symptoms. In this study, 93% of the participants had never suffered from MBDs, and Taiwan is a region with a relatively low prevalence of infectious diseases. Some migrant workers may think that since they have never contracted the disease in the Philippines in the past, the chances of contracting the disease in Taiwan is even lower.

The purpose of all health education is nothing more than to promote preventive practices. This study found that knowledge provides an important contribution to preventive practices. This is consistent with the literature discussing the knowledge and prevention behaviors for other MBDs [14,17,18]. Since most migrant workers had lower levels of education and had limited health-related knowledge, improving migrant workers’ awareness of infectious diseases is undoubtedly one of the most effective and convenient strategies to prevent the occurrence of diseases.

Perceived severity is also an important factor in influencing preventive behavior. If migrant workers recognize that dengue fever or malaria is a serious threat to their health or life, they will take preventive measures against the disease. Other studies also demonstrated similar results [11,14]. Therefore, the contents of health education promotions for migrant workers need to appropriately emphasize the serious consequences of suffering from diseases, so that we can increase the possibility of people adopting preventive behaviors. In fact, when migrant workers realize that there will be serious consequences once contracting a disease, the perceived benefits will increase. This relationship was also confirmed by the path analysis, which promoted the occurrence of preventive practices. Similarly, this study also pointed out that there was a negative correlation between perceived barriers and preventive practice, indicating that if the perception of barriers is reduced through health education, it will effectively increase the chance of migrant workers taking preventive practices. This result has been supported by past research [17].

When interpreting the results of this study, we need to take several methodological limitations into consideration. First, this research only focuses on migrant workers from the Philippines. Therefore, we cannot generalize this result to migrant workers of other nationalities. In addition, due to time, labor, and other constraints, the post-test was conducted immediately after the HEI, and we cannot further assess the long-term effect.

## 5. Conclusions

The preliminary conclusions of this study point out that HEIs for migrant workers should focus on the improvement of knowledge, perceived severity, perceived benefits, and perceived barriers in order to effectively produce preventive practices. Relevant information can be transmitted through the media (especially television). Additionally, multi-language health and education graphic promotions, or preventive lectures can be placed in the routine medical examinations of the foreign migrant workers. This will promote the health of foreign migrant workers and reduce the outbreak and spread of diseases.

## Figures and Tables

**Table 1:** Sociodemographic characteristics and cues to action of the participants of the present study (*n* = 291).

Variable		*n*	%
Gender	Male	63	21.6
	Female	228	78.4
Age	20–24	50	17.2
	25–29	115	39.5
	30–34	77	26.5
	35–39	27	9.3
	40–44	22	7.6
Highest educational attainment	Primary education	18	6.2
	Secondary education	80	27.5
	Higher education	193	66.3
First time to Taiwan	Yes	172	59.1
	No	119	40.9
Duration of stay in Taiwan	0–2 years	69	23.7
	2–4 years	128	44.0
	4–6 years	45	15.5
	6–8 years	23	7.9
	8–10 years	18	6.2
	More than 10 years	8	2.7
Type of job in Taiwan	Manufacturing	266	91.4
	Human health	22	7.6
	Construction	3	1.0
Mandarin proficiency	Not	61	21.0
	Listen	52	17.9
	Listen and speak	175	60.1
	Read and listen and speak	3	1.0
Average monthly income (New Taiwan Dollar, NTD) ^a^	10–20 thousand	156	53.6
	20–40 thousand	135	46.4
	40–50 thousand	2	0.7
Where do you get information about mosquito-borne diseases? (tick all that apply)	Original knowledge	68	23.4
	Television	148	50.9
	Hospital	109	37.5
	Books/newspapers	84	28.9
	Clinic	38	13.1
	Network	38	13.1
Who gives you information about mosquito-borne diseases? (tick all that apply)	Taiwanese friends	39	13.4
	Family	129	44.3
	Doctor/nurse	130	44.7
	Filipino friends	82	28.2
Have you had a mosquito-borne disease before?	Yes	18	6.2
	No	273	93.8
Has anyone in your family been infected with a mosquito-borne disease before?	Yes	38	13.1
	No	253	86.9

^a^ 1 NTD = 0.035 USD at the time of the study.

**Table 2:** Correct number and percentage for knowledge items about mosquito-borne diseases and item scores of health beliefs and preventive practices toward mosquito-borne diseases before and after implementation of the health education program (*n* = 291).

Knowledge/Health Beliefs/Preventive Practices	Pre-Test		Post-Test		*p*
**Knowledge**	***n***	**%**	***n***	**%**	
1. Dengue fever is transmitted by mosquitoes	274	94.2	289	99.3	0.100
2. Drinking unsafe water and eating raw food will result in malaria infections	46	15.8	218	74.9	<0.0001
3. Place a tight lid on containers used for water storage to avoid vector breeding	241	82.8	288	99.0	<0.0001
4. Vaccination is the best way to prevent malaria	61	20.9	210	72.2	<0.0001
5. Malaria may be transmitted to the fetus through the mother	162	55.7	274	94.2	<0.0001
6. Using repellents containing DEET (N, N–diethyl–m–toluamide) on clothing and exposed skin can prevent dengue fever infection	166	57.0	259	89.0	<0.0001
7. Putting on a mask prevents malaria	100	34.4	238	81.8	<0.0001
8. Wash hands frequently to avoid spreading dengue fever	68	23.4	228	78.4	<0.0001
9. Stagnant water in pots will let the vector breed	233	80.1	249	85.6	0.090
10. If you are bitten by an animal you might get malaria	93	32.0	179	61.5	<0.0001
11. For people, getting dengue fever one time can provide immunity for life	89	30.6	202	69.4	<0.0001
12. Headache and muscle ache are the common symptoms of malaria	223	76.6	279	95.9	<0.0001
13. Dengue fever may trigger dengue hemorrhagic fever	249	85.6	272	93.5	0.003
14. Dengue virus will not spread to a person with a strong immune system when bitten by an infected mosquito	109	37.5	195	67.0	<0.0001
15. The symptoms of early malaria are fever and chills	256	88.0	282	96.9	<0.0001
16. Prevent mosquito bites by wearing long-sleeve shirts	203	69.8	274	94.2	<0.0001
17. If you have any symptoms, you should seek medical help and inform the doctor of your travel history	262	90.0	264	90.7	0.890
18. All mosquito-borne diseases are caused by dengue virus	49	16.8	176	60.5	<0.0001
19. Mosquito-borne diseases are spread from person to person	128	44.0	182	62.5	<0.0001
20. Outbreaks of mosquito-borne diseases occur in the southeast area	140	48.1	244	83.8	<0.0001
**Perceived susceptibility**	Mean	SD	Mean	SD	
1. I have a high risk of getting a mosquito-borne disease	4.00	0.91	4.08	0.96	0.324
2. I have a high risk of contacting a patient who has a mosquito-borne disease	3.91	0.95	3.92	0.96	0.894
**Perceived severity**	Mean	SD	Mean	SD	
1. If I get a mosquito-borne disease, I will need hospitalization	3.69	1.02	4.60	0.61	<0.0001
2. If I get a mosquito-borne disease, I will die	3.91	1.06	4.59	0.68	<0.0001
3. If I get a mosquito-borne disease, I will suffer	3.89	0.87	3.96	0.80	0.346
4. If I get a mosquito-borne disease, I will not be able to work	3.84	0.87	3.90	0.80	0.326
5. If I get a mosquito-borne disease, it will affect my relationships with other people	3.62	1.03	3.87	0.79	<0.0001
6. If I get a mosquito-borne disease, it might affect my visa in the future	3.61	1.04	3.84	0.78	<0.0001
**Perceived benefit**	Mean	SD	Mean	SD	
1. Taking measures to prevent diseases can keep me healthy	4.07	0.95	4.61	0.63	<0.0001
2. Taking measures to prevent diseases can prevent hospitalization	3.98	1.02	4.60	0.60	<0.0001
3. Taking measures to prevent diseases can reduce the cost of medical expenses	4.03	0.98	4.11	0.76	0.243
4. Taking measures to prevent diseases can avoid spreading a disease to family or friends	3.96	1.02	4.11	1.92	0.231
5. My friends and colleagues use insecticide sprays and bed nets	3.01	0.97	4.01	0.79	0.392
**Perceived barriers**	Mean	SD	Mean	SD	
1. I don’t know where to find information about mosquito-borne diseases	2.63	1.39	2.55	1.39	0.166
2. I am healthy, I don’t need to take preventive measures	2.76	1.27	2.51	1.38	0.010
3. Colleagues or friends do not think it is necessary to prevent the spread of infectious diseases	2.89	1.23	2.69	1.25	0.034
4. It bothers me to take prevention measures	3.02	1.28	2.79	1.25	0.017
5. Prevention practices will take a lot of money	2.97	1.29	2.91	1.24	0.568
6. Because of a language barrier, I can’t get information about preventive measures	2.98	1.26	2.89	1.24	0.325
7. I have concerns about undesirable hazards relating to mosquito coils or insecticide sprays	3.32	1.18	2.98	1.26	<0.0001
**Preventive practices**	Mean	SD	Mean	SD	
1. Used mosquito net	3.55	1.17	4.71	0.59	<0.0001
2. Removed sources of stagnant water from pots or tires	3.69	1.21	4.74	0.53	<0.0001
3. Removed rubbish blocking the drains	3.63	1.26	4.38	0.71	<0.0001
4. Used mosquito coils	3.48	1.28	4.20	0.78	<0.0001
5. Used insecticide sprays	3.52	1.30	4.21	0.77	<0.0001
6. Wore long sleeves when going outside	3.40	1.30	4.27	0.77	<0.0001
7. Used screens on doors and windows	3.52	1.36	4.27	0.79	<0.0001
8. Told my colleagues or friends the importance of preventing mosquito-borne diseases	3.55	1.40	4.41	0.80	<0.0001

*p* for McNemar Chi-square test among knowledge items and for paired *t* test among items of health beliefs and preventive practices. SD: standard deviation.

**Table 3:** Paired *t* test for total scores of knowledge, health beliefs, and preventive practices toward mosquito-borne diseases before and after the implementation of the health education program.

		Mean	SD	DF	Paired *t*	*p*
Knowledge	Pre-test	10.70	2.65	290	−27.00	<0.0001
	Post-test	16.50	2.59			
Perceived susceptibility	Pre-test	7.91	1.67	290	−0.60	0.545
	Post-test	8.00	1.83			
Perceived severity	Pre-test	22.56	4.62	290	−6.64	<0.0001
	Post-test	24.75	3.66			
Perceived benefit	Pre-test	18.79	3.66	290	−8.80	<0.0001
	Post-test	21.44	3.35			
Perceived barriers	Pre-test	20.58	6.82	290	2.38	0.018
	Post-test	19.31	8.38			
Preventive practices	Pre-test	28.32	9.48	290	−11.09	<0.0001
	Post-test	35.19	4.49			

SD: standard deviation; DF: degree of freedom.

## Data Availability

The datasets used and analyzed during the current study are available from the corresponding author on reasonable request.

## Supplementary Materials

The following supporting information can be downloaded at: https://www.mdpi.com/article/10.3390/ijerph19020787/s1, S1: Questionnaire A and Questionnaire B.

## Abbreviations

MBD	mosquito-borne disease
HEI	health education intervention
WHO	World Health Organization
HBM	health belief model
TCDC	Taiwan Centers for Disease Control
NTD	New Taiwan Dollar
