# Supplementary material for: Factors That Prevent Mosquito-Borne Diseases among Migrant Workers in Taiwan: Application of the Health Belief Model in a Church-Based Health Promotion Study
Source: Int J Environ Res Public Health. 2022 Jan 11;19(2):787. doi: 10.3390/ijerph19020787 (PMC8776191; doi:10.3390/ijerph19020787)
Supplement: Supplementary file 1 [file ijerph-19-00787-s001.zip › ijerph-1521181-supplementary.pdf]

**Questionnaire A** V1.0 Date 2018/05/03

**A. Basic information**

1. Gender：□①Male □②Female
2. Age(years)：
   □①20-24 □②25-29 □③30-34 □④35-39 □⑤40-44 □⑥45-49 □⑦above 50
3. Highest educational attainment：
   □①No formal education□②primary education □③secondary education
   □④more than secondary education
4. First time to Taiwan：□①Yes □②No
5. Duration of stay in Taiwan：(totally)
   □①0-2 years □②2-4years □③4-6years□④6-8years
   □⑤8-10years □⑥more than 10 years
6. Type of job in Taiwan：
   □①Manufacturing □②Human health □③Agriculture and forestry
   □④Construction □⑤Fishing □⑥Forestry □⑦Mining
7. Chinese level：
   □①Not □②listen □③listen and speak □④read and listen and speak
8. Average monthly income：(Taiwan dollars)
   □①10-20 thousand □②20-40 thousand □③40-50 thousand □④more than 50 thousand

**B. Knowledge about mosquito-borne diseases**

1. Dengue fever transmitted by mosquito.
   □①True □②Fales □③Don't know
2. Drink unsafe water and eating raw food will infect malaria.
   □①True □②Fales □③Don't know
3. Place a tight lid on containers used for water storage to avoid vector breeding.
   □①True □②Fales □③Don't know
4. Vaccination is the best way to prevent malaria. □①True □②Fales □③Don't know
5. Malaria may be transmitted to fetus through the mother. □①True □②Fales □③Don't know
6. Use repellents containing DEET (N, N–diethyl–m–toluamide) on clothing and exposed skin can prevent dengue fever infection. □①True □②Fales □③Don't know
7. Put mask to prevent malaria.□①True □②Fales □③Don't know
8. Washing hand frequently to avoid spreading dengue fever. □①True □②Fales □③Don't know
9. Stagnant water in pots will let vector breeding. □①True □②Fales □③Don't know
10. Bitten by animals maybe get malaria. □①True □②Fales □③Don't know
11. For people, getting dengue fever once provides immunity for life.
    □①True □②Fales □③Don't know
12. Headache and muscle ache are the common symptoms of malaria.
    □①True □②Fales □③Don't know
13. Dengue fever may trigger dengue hemorrhagic fever. □①True □②Fales □③Don't know
14. Dengue virus will not spread to a person with a strong immune system when bitten by an infected mosquito. □①True □②Fales □③Don't know

15. The symptoms of early malaria have fever and chillness.
□①True □②Fales □③Don’t know
16. Against mosquito bites through wearing long-sleeve shirts.
□①True □②Fales □③Don’t know
17. If any symptoms, should seek medical and inform doctor the history of travel.
□①True □②Fales □③Don’t know
18. All Mosquito-borne diseases are caused by dengue virus. □①True □②Fales □③Don’t know
19. Mosquito-borne diseases are spread from person to person.
□①True □②Fales □③Don’t know
20. Mosquito-borne diseases outbreak in southeast area. □①True □②Fales □③Don’t know

**C. Cues to action of mosquito-borne diseases**

1. Where are you get information about mosquito-borne diseases？(you can choice multiple)
□①No □②original knowledge □③television □④Hospital
□⑤Books / newspapers / magazines □⑥clinic □⑦network □⑧other_____
2. Who are told you information about mosquito-borne diseases？(you can choice multiple)
□①No □②Taiwan’s friends □③family □④doctor/nurse
□⑤Philippine’s friends □⑥other______
3. Have you been got Mosquito-borne diseases before？ □①Yes □②No
4. Does your family have been infected with mosquito-borne diseases before？ □①Yes □②No

**D. Health beliefs model**

1. Mosquito-borne diseases perceived susceptibility

| I have a high risk of….. | very impossible | impossible | Neutral | possible | very possible |
|---|---|---|---|---|---|
| (1) getting mosquito-borne diseases | | | | | |
| (2) contact patient who getting mosquito-borne diseases | | | | | |

2. Mosquito-borne diseases perceived severity

| When get mosquito-borne diseases, | very not serious | not serious | Neutral | serious | very serious |
|---|---|---|---|---|---|
| (1) I will need hospitalization | | | | | |
| (2)I will dead | | | | | |
| (3) I will suffering | | | | | |
| (4)I can’t work | | | | | |
| (5) I will affect my relationship with other people. | | | | | |
| (6) maybe affects my visa in future. | | | | | |

3. Mosquito-borne diseases perceived benefit.

| Take measures to prevent diseases... | totally disagree | partly disagree | Neutral | partly agree | totally agree |
|---|---|---|---|---|---|
| (1) can keep healthly | | | | | |
| (2) can prevent hospitalization | | | | | |
| (3)can reduce the cost of medical expenses | | | | | |

| | | | | | |
|---|---|---|---|---|---|
| (4)can avoid spreading to family or friends | | | | | |
| (5) my friends and colleague were used insecticide sprays and bed net | | | | | |

4.Mosquito-borne diseases prevention barriers

| | totally disagree | partly disagree | Neutral | partly agree | totally agree |
|---|---|---|---|---|---|
| (1)Don't know where has information about mosquito-borne diseases | | | | | |
| (2)I am healthy, don't need to take preventive measures | | | | | |
| (3) Colleagues or friends do not think it necessary to prevent the spread of infectious disease | | | | | |
| (4) It bothers me to take prevention measures | | | | | |
| (5)Prevention practices will take much money | | | | | |
| (6)Because language barriers,I can't get information about preventive measures | | | | | |
| (7)Concerns about undesirable hazards relating to mosquito coil or insecticide sprays | | | | | |

5. Prevetion of mosquito-borne diseases

| Did you have the following behaviors? | very impossible | impossible | Neutral | possible | very possible |
|---|---|---|---|---|---|
| (1) Used mosquito net | | | | | |
| (2)Removed sources of stagnant water from pots or tire | | | | | |
| (3) Removed rubbish blocking the drains. | | | | | |
| (4)Used mosquito coils | | | | | |
| (5) Used insecticide sprays | | | | | |
| (6)Wore long sleeves when go outside | | | | | |
| (7)Used screens on doors and windows. | | | | | |
| (8) Told my colleagues or friends the importance of prevent mosquito-borne diseases | | | | | |

**Questionnaire B** V1.0 Date 2018/05/03

**A. Knowledge about mosquito-borne diseases**

21. Dengue fever transmitted by mosquito.
□①True □②False □③I don't know
22. Drink unsafe water and eating raw food will infected malaria
□①True □②False □③I don't know
23. Place a tight lid on containers used for water storage to avoid vector breeding
□①True □②False □③I don't know
24. Vaccination is the best way to prevent malria □①True □②False □③I don't know
25. Malaria may be transmitted to fetus through the mother □①True □②False □③I don't know
26. Use repellents containing DEET (N, N–diethyl–m–toluamide) on clothing and exposed skin can prevent dengue fever infection □①True □②False □③I don't know
27. Put mask to prevent malaria□①True □②False □③I don't know
28. Washing hand frequently to avoid spreading dengue fever □①True □②False □③I don't know
29. Stagnant water in pots will let vector breeding □①True □②False □③I don't know
30. Bitten by animals maybe get malaria □①True □②False □③I don't know
31. For people, getting dengue fever once provides immunity for life.
□①True □②False □③I don't know
32. Headache and muscle ache are the common symptoms of malaria
□①True □②False □③I don't know
33. Dengue fever may trigger dengue haemorrhagic fever □①True □②False □③I don't know
34. Dengue virus will not spread to a person with a strong immune system when bitten by an infected mosquito □①True □②False □③I don't know
35. The symptoms of early malaria has fever and chillness
□①True □②False □③I don't know
36. Against mosquito bites through wearing long-sleeve shirts
□①True □②False □③I don't know
37. If any symptoms,should seek medical and inform doctor the history of travel
□①True □②False □③I don't know
38. All Mosquito-borne diseases are caused by dengue virus □①True □②False □③I don't know
39. Mosquito-borne diseases are spread from person to person
□①True □②False □③I don't know
40. Mosquito-borne diseases outbreak in southeast area □①True □②False □③I don't know

**B. Health beliefs model**

1. Mosquito-borne diseases perceived susceptibility

| I have a high risk of….. | very impossible | impossible | Neutral | possible | very possible |
|---|---|---|---|---|---|
| (1) getting mosquito-borne diseases | | | | | |
| (2) contact patient who getting mosquito-borne diseases | | | | | |

2. Mosquito-borne diseases perceived severity

| When get mosquito-borne diseases, | very not serious | not serious | Neutral | serious | very serious |
|---|---|---|---|---|---|
| (1) I will need hospitalization | | | | | |
| (2)I will dead | | | | | |
| (3) I will suffering | | | | | |
| (4)I can't work | | | | | |
| (5)I will affect my relationship with other people. | | | | | |
| (6) maybe affects my visa in future. | | | | | |

3. Mosquito-borne diseases perceived benefit.

| Take measures to prevent diseases ….. | totally disagree | partly disagree | Neutral | partly agree | totally agree |
|---|---|---|---|---|---|
| (1) can keep healthly | | | | | |
| (2) can prevent hospitalization | | | | | |
| (3)can reduce the cost of medical expenses | | | | | |
| (4)can avoid spreading to family or friends | | | | | |
| (5) my friends and colleague were used insecticide sprays and bed net | | | | | |

4.Mosquito-borne diseases prevetion barriers

| | totally disagree | partly disagree | Neutral | partly agree | totally agree |
|---|---|---|---|---|---|
| (1)I don't know where has information about mosquito-borne diseases | | | | | |
| (2)I am healthy, don't need to take preventive measures | | | | | |
| (3) Colleagues or friends do not think it necessary to prevent the spread of an infectious disease | | | | | |
| (4) It bothers me to take prevention measures | | | | | |
| (5)Prevention practices will take much money | | | | | |
| (6)Because language barriers,I can't get information about preventive measures | | | | | |
| (7)Concerns about undesirable hazards relating to mosquito coil or insecticide sprays | | | | | |

5. Mosquito-borne diseases prevention self-efficacy

| How confident are you to perform the following behaviors? | completely confident | Mostly confident | partly confident | Little confident | Not at all confident |
|---|---|---|---|---|---|
| (1) Use mosquito net | | | | | |
| (2)Remove sources of stagnant water from pots or tire | | | | | |
| (3) Remove rubbish blocking the drains. | | | | | |
| (4)Use mosquito coils | | | | | |
| (5) Use insecticide sprays | | | | | |
| (6)Wear long sleeves when go outside | | | | | |
| (7)Use screens on doors and windows. | | | | | |
| (8) Tell my colleagues or friends the importance of prevent mosquito-borne diseases | | | | | |

# *Research Informed Consent Form*

本院案號：CS18132

版本：V1.0　　，日期：2018/05/03

**Project:** The factors of prevention mosquito-borne diseases among migrant workers in Taiwan: Application of the Health Belief Model

## I. Introduction：

You are being invited to participate in this research. This form provides information related to this study. The researcher will explain the content of this study to you and answer any questions you may have. Please do not sign this consent form until all of your questions have been answered satisfactorily. If you are willing to participate in this study, this document will be considered as the record of your consent. You can withdraw from the study at any time without any reason, even after you have given consent.

Research institute:Chung Shan Medical University

Principal investigator:Hao-Jan,Yang　Title:Professor　TEL:04-24730022# 12109

Researcher:Yu-Shan,Tai　Title:Student　TEL:0926090243

Emergency contact person:Yu-Shan,Tai　TEL:0926090243

1. Research objective:

   evaluate the connections of foreign workers' knowledge, health beliefs and behaviors about mosquito-borne diseases,and the outcomes of health education intervention.

2. Research methods:

   First fill out questionnaire A(pre-test),and read the handout forms,then fill out questionnaire B(post-test).

3. Research participants:300 people are needs in this research.
   (1) inclusion criteria: 20 years old and above and Philippines' foreign workers.
   (2) exclusion criteria: did not participate in complete research.
4. Research time:about 30-40minutes.

## II. Confidentiality of subjects personal information：

We will abide by the law to keep the confidentiality of any your personal private information, and will not disclose it. In the event that study results are published, your identification will continue to be kept confidential. You also understand that by signing this consent form, you are approving direct use of your original records by the monitors, auditors, (Chung Shan Medical University hospital IRB) and the competent authorities, in order to ensure that the study is conducted and data are collected in accordance with applicable laws.

The research staff will assign you with a research code, and this code will not show any identifiable information such as your name. Your identification will continue to be kept confidential and the

questionnaire will be stored destroyed by researcher after study results are published.

## III. Anticipated commercial benefit(s) derived from the study:

This research is not expected to derive any patents or other commercial interests.

## IV. Rights of the subject：

(1) We will give you a gift (worth 50NTD).

(2) During the study, if you have any questions about the nature of the study or any concerns about your rights as a patient, or suspect that you have suffered injury as a result of participating in this research, please contact the Chung Shan Medical University hospital IRB to request for consultation. The telephone number is 04-24739595 ext. 04-35073516. E-mail:irb@csh.org.tw.

(3) if you have any questions about the study, please contact researcher Yu-Shan,Tai TEL: 0926090243.

(4) This consent form is made in duplicate. The research: Yu-Shan,Tai has given you a copy of the consent form and has fully explained the nature and purpose of this research

## V. Signature：

(1) The researcher has explained in detail the nature and objectives of the above research method in this consent

Signature of Principal Investigator/Sub-investigator:___________________

Date: ______(Month) ________(Day), __________(Year)

(2) I fully understand the research method mentioned above and my questions about the study have been answered in full detail. I agree to participate in this research voluntarily and will hold a duplicate of the Informed Consent Form.

Signature of the Subject:_________________________

Date: ______(Month) ________(Day), __________(Year)
